# Supplementary material for: Application and utility of a clinical framework for spinally referred neck-arm pain: A cross-sectional and longitudinal study protocol
Source: PLoS One. 2020 Dec 28;15(12):e0244137. doi: 10.1371/journal.pone.0244137 (PMC7769468; doi:10.1371/journal.pone.0244137)
Supplement: S1 File — (DOCX) [file pone.0244137.s001.docx]

| **CLINICAL EXAMINATION FORM** |
| --- |

**ID:**

**DOB:**

**Date: Age: Sex: Profession:**

**Dominant side: Height: Body Weight: BMI:**

**Main Problem:**


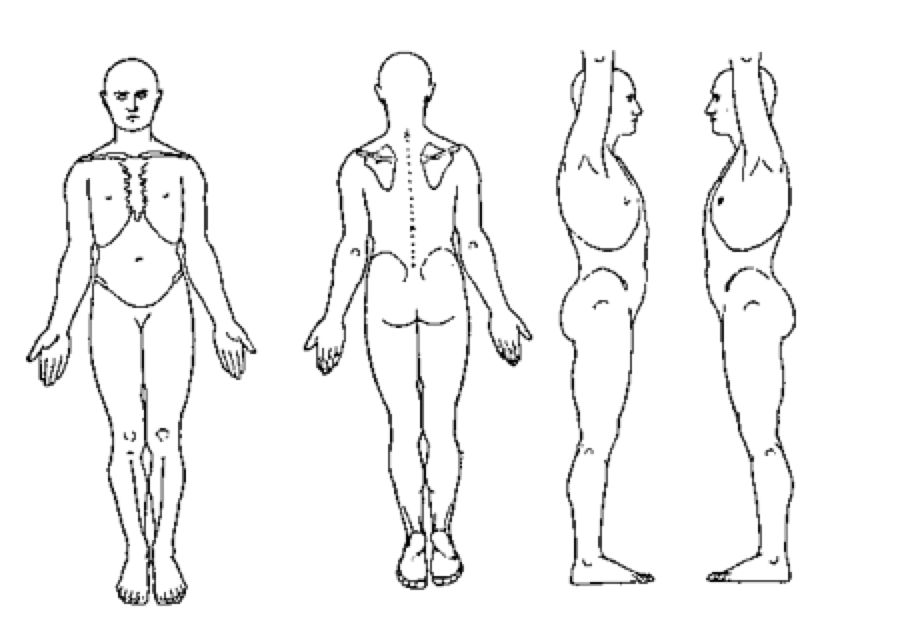


**Current Hx:**

**Past Hx:**

**Pain behaviour:**

Aggravating factors:

Ease factors:

Mechanical pain behaviour:

Spontaneous pain behaviour:

Painful cold:

**24h:**

N:

Do you wake up tired and not recovered? ‘Never’ ‘seldom’ ‘often or usual’ ‘always’

Sleep quality:

_______________________________________________

0 10

Good sleep Poor sleep

AM:

During the day:

End of day:

**General health:**

Comorbidities/fracture/Trauma/Op:

Fatigue: Are you generally tired over the day? ‘Never’ ‘seldom’ ‘often or usual’ ‘always’

Diabetes:

Thyroid disease:

Rheumatoid arthritis:

B12:

Handling or working with toxins:

X-Ray, MRI etc.:

Weight loss:

Spinal cord symptoms:

Vertebral artery signs:

Reaction to coughing/sneezing:

Medication:

| **Name** | **Dosage** | **Duration of intake** | **Effect** |
| --- | --- | --- | --- |
|  |  |  |  |
|  |  |  |  |
|  |  |  |  |
|  |  |  |  |
|  |  |  |  |
|  |  |  |  |

| **PHYSICAL EXAMINATION** |
| --- |

**pp (NRS):**

**Inspection:**

**Functional demonstration:**

**Active movements:**

| **Cervical spine** | **Range, Symptom, Quality of movement** | **Shoulders** | **Range, Symptom, Quality of movement** |
| --- | --- | --- | --- |
| Flexion: |  | Flexion: |  |
| Extension: |  | Abduction: |  |
| Rotation right: |  | Abduction + cervical contralateral Lateral flexion: |  |
| Rotation left: |  | Abduction + hand dorsal extension: |  |
| Lateral flexion right: |  |  | |
| Lateral flexion left: |  |  |  |

**Spurling’s Test (Lateral Flexion, Rotation, Compression.):**

**Right:**

**Left:**

**Neurological examination**

| **Strength**  **Rating 5/5=√** | **Left** | **Right** |
| --- | --- | --- |
| Scapula Elev. (C4) |  |  |
| Shoulder ABD (C5) |  |  |
| Elb. Flex. (C5/6) |  |  |
| Elb. Ext. (C6/7) |  |  |
| Hand Ext. (C7) |  |  |
| Thumb Ext. (C8)  Finger Flex. |  |  |
| Interossei (T1) |  |  |
| others |  |  |

| **Reflex** | **Hyperreflexia/Clonus: ++++**  **Increased: +++**  **Normal: ++**  **Reduced: +**  **Absent: -** | |
| --- | --- | --- |
|  | **Left** | **Right** |
| BICEPS |  |  |
| TRICEPS |  |  |
| BRACHIORADIAL |  |  |
| PRONATOR QUA. |  |  |

| **Sensation** | **Intensity**  **N** = normal  **Less** = Hypoaesthesia  **Loss** = Analgesia  **Increased** = Hyperaesthesia | **Quality**  **N** = Normal  **C = Changed** (Paraesthesia,  Dysaesthesia (unpleasant), Allodynia) |
| --- | --- | --- |

|  | **Dermotome C4 – T1** | | | |
| --- | --- | --- | --- | --- |
|  | **Left** | | **Right** | |
|  | Intensity | Quality | Intensity | Quality |
| Soft touch (cotton wool) |  |  |  |  |
| Pin Prick |  |  |  |  |

| **Maximal pain area:** | | | | |
| --- | --- | --- | --- | --- |
|  | **Left** | | **Right** | |
|  | Intensity | Quality | Intensity | Quality |
| Soft touch  (cotton wool) |  |  |  |  |
| Pin Prick |  |  |  |  |
| Cold (Coin) |  |  |  |  |
| Warm (Coin) |  |  |  |  |
| Ice cube Test |  |  |  |  |
| Vibration |  |  |  |  |
| **Second pain area:** | | | | |
| Soft touch  (cotton wool) |  |  |  |  |
| Pin Prick |  |  |  |  |
| Cold (Coin) |  |  |  |  |
| Warm (Coin) |  |  |  |  |
| Ice cube Test |  |  |  |  |
| Vibration |  |  |  |  |

| **Others** | **Left** | **Right** |
| --- | --- | --- |
| BABINSKI |  |  |
| CLONUS |  |  |
| Gait |  |  |
| BALANCE  Single leg stand  Romberg Test |  |  |
| COORDINATION  Tandem walking |  |  |

**Upper Limb Neurodynamic Tests:**

**Nerv palpation:**

**Right Left**

Median nerve:

Radial nerve:

Ulnar nerve:

**Passive movements cervical spine**

Passive Physiological Intervertebral Movements cervical spine:

Passive Accessory Intervertebral Movements cervical spine:

Central, unilateral (with angulations) Cervical 1 – 7

**Palpation (Triggerpoints):**

**Scalenus muscle right: left:**

**Pectoralis minor muscle right: left:**

**Subclavius muscle right: left**

**Infraspinatus muscle right: left:**

**M. levator scap right: left:**

**Upper trapezius right: left:**
