# Supplementary material for: Application and utility of a clinical framework for spinally referred neck-arm pain: A cross-sectional and longitudinal study protocol
Source: PLoS One. 2020 Dec 28;15(12):e0244137. doi: 10.1371/journal.pone.0244137 (PMC7769468; doi:10.1371/journal.pone.0244137)
Supplement: Attachment — Submitted filename: Response to Reviewer Comments.docx [file pone.0244137.s003.docx]

**Response to Reviewer Comments**

**Study Protocol Title:**

**Application and utility of a clinical framework for spinally referred neck-arm pain : A cross-sectional and longitudinal study protocol**

**Thank you very much to the editor and all reviewers for reviewing our study protocol and for the helpful comments.**

| Comment | Response |
| --- | --- |
| 1. The title - As it stands, the changed title might benefit from some simplification to: "Application and utility of a clinical framework for spinally referred neck-arm pain: A cross-sectional and longitudinal study protocol" | Thank you for your comment.  We changed the title to: Application and utility of a clinical framework for spinally referred neck-arm pain: A cross-sectional and longitudinal study protocol |
| 2. In addition, the word "protocol" does not feature in the abstract which might be misleading | Thank you very much. We added the following sentence in the Method section of the abstract part L 35:  We describe a study protocol. |
| 3. Ethics - first sentence would be improved by stating "The study has received ethical approval form the … " | We added your recommendation, Page 7 L 160-161: The study has received ethical approval from the ~~was approved by the~~ Ethics Committee of the University of Applied Sciences Osnabrück (HSOS/2019/2/2) |
| 4. Quantitative Sensory Testing (QST) section (L327 of tracked changes version), there is a "the" missing before "German ..." | Thank you. We added the missing word page 12 L289-290:  Standardized QST will be performed according to the reliable QST protocol of the German Research Network on Neuropathic Pain (DFNS) |
| 5. L364 of tracked changes version, Lime survey - is propitiatory software/a company that, if so, would benefit from having company details/version nos included. | We added the missing information on page 13 L 326:  The three, six and 12 months follow-up will be conducted via postal hard copy and lime survey (Version: 3.22.210 + 200804). |
| 6. L387 of tracked changes version, adding company details/version nos to the SPSS and R allusion would be more complete(less...) | We added the missing informations page 14 L349:  All data analysis will be performed with SPSS (Version: 26) and R (Version: 3.6.3.). |
